# Genetic Analysis of Reduced *γ*-Tocopherol Content in Ethiopian Mustard Seeds

**DOI:** 10.1155/2016/7392603

**Published:** 2016-05-05

**Authors:** Elena García-Navarro, José M. Fernández-Martínez, Begoña Pérez-Vich, Leonardo Velasco

**Affiliations:** Institute for Sustainable Agriculture (IAS-CSIC), Alameda del Obispo s/n, 14004 Córdoba, Spain

## Abstract

Ethiopian mustard (*Brassica carinata* A. Braun) line BCT-6, with reduced *γ*-tocopherol content in the seeds, has been previously developed. The objective of this research was to conduct a genetic analysis of seed tocopherols in this line. BCT-6 was crossed with the conventional line C-101 and the F_1_, F_2_, and BC plant generations were analyzed. Generation mean analysis using individual scaling tests indicated that reduced *γ*-tocopherol content fitted an additive-dominant genetic model with predominance of additive effects and absence of epistatic interactions. This was confirmed through a joint scaling test and additional testing of the goodness of fit of the model. Conversely, epistatic interactions were identified for total tocopherol content. Estimation of the minimum number of genes suggested that both *γ*- and total tocopherol content may be controlled by two genes. A positive correlation between total tocopherol content and the proportion of *γ*-tocopherol was identified in the F_2_ generation. Additional research on the feasibility of developing germplasm with high tocopherol content and reduced concentration of *γ*-tocopherol is required.

## 1. Introduction

 Oilseed crops contain high levels of tocopherols, which prevent lipid oxidation and subsequently contribute to improve seed longevity [1]. Due to their nonpolar nature, tocopherols become part of the seed oil after oil extraction, where they play a key role as* in vitro* and* in vivo* antioxidants.* In vitro*, tocopherols are the main compounds that protect the oil from lipid peroxidation that results in off flavors and reduced shelf life [2].* In vivo* activity of dietary tocopherols is exerted in the human or animal body after they are consumed in the diet or in vitamin supplements, where they protect the cells from oxidative stress [3]. Naturally occurring tocopherols exist in four forms termed as *α*-, *β*-, *γ*-, and *δ*-tocopherol. They differ not only in molecular structure, but also in biological effectiveness; whereas *α*-tocopherol is the tocopherol form with higher biological value as vitamin E [4], *γ*- and *δ*-tocopherol exhibit higher* in vitro* antioxidant activity, particularly at high temperatures [5, 6].

Ethiopian mustard (*Brassica carinata* A. Braun) is a vegetable and oilseed crop mainly cultivated in Ethiopia, where it plays an important role in agricultural systems and human nutrition [7]. The crop has potential for other areas as a source of vegetable oil for either industrial or food uses due to the availability of germplasm with different fatty acid profiles, including high erucic acid and low erucic acid types [8]. The tocopherol fraction in seeds of the genus* Brassica*, which includes the major oilseed crop rapeseed/canola (*Brassica napus* L.), is predominantly made up of varying proportions of *α*- and *γ*-tocopherol, with the latter being in general the predominant tocopherol form [9, 10]. Only in a few cases has germplasm with predominance of *α*-tocopherol been reported, for example, in* B. rapa* [9] and* B. carinata* [9, 11]. The relative concentrations of *α*- and *γ*-tocopherol are mainly determined by a *γ*-tocopherol methyltransferase (*γ*-TMT), which catalyses a methylation step from *γ*-tocopherol to *α*-tocopherol [12]. Transgenic lines with elevated proportion of *α*-tocopherol of about sixfold over the control plants have been developed in* B. juncea* Czern. & Coss. by overexpression of *γ*-TMT [13].

Several studies have been conducted on the mode of inheritance of *α*- and *γ*-tocopherol contents in* Brassica* spp. In rapeseed, studies using factorial [14] and diallel [15] mating designs concluded that the contents of both tocopherols as well as the total tocopherol content were mainly controlled by additive gene effects. In addition to additive effects, significant additive × environment interactions have been also detected [16]. A study in the same crop, using three doubled haploid populations of different genetic origin, concluded that G × E effects were larger than genetic effects, resulting in low broad-sense heritabilities from 0.23 to 0.46 for *α*-tocopherol content, from 0.36 to 0.50 for *γ*-tocopherol content, and from 0.34 to 0.48 for total tocopherol content [17]. Slightly higher heritability values (0.58 for *α*-tocopherol content, 0.43 for *γ*-tocopherol content, and 0.53 for total tocopherol content) were reported in a study of a rapeseed population of 25 F_1_-derived doubled haploid lines [18]. Contrastingly, greater heritability values were estimated in two sets of rapeseed accessions, 0.77 and 0.89 for *α*-tocopherol content, 0.78 and 0.82 for *γ*-tocopherol content, and 0.62 and 0.78 for total tocopherol content [19]. Similarly, the evaluation of a segregating F_1_-derived doubled haploid population of 202 lines revealed broad-sense heritability values of 0.81 for *α*-tocopherol content, 0.85 for *γ*-tocopherol content, and 0.65 for total tocopherol content [20]. In Ethiopian mustard, germplasm with very high relative concentration of *γ*-tocopherol (ca. 75% of total tocopherols compared to 44% in conventional germplasm) was identified. The trait was found to be controlled by partially recessive alleles at a single locus [11]. Germplasm with increased relative concentration of *α*-tocopherol, around 70% of total tocopherols, was also reported in the same research. Such a high concentration of *α*-tocopherol has not been identified in other* Brassica* spp., including transgenic* B. juncea* lines that had a maximum concentration of *α*-tocopherol around 60% [13]. Increased *α*-tocopherol concentration in Ethiopian mustard was the result of reduced *γ*-tocopherol content rather than an increase in *α*-tocopherol accumulation [11].

The objective of this research was to conduct a genetic analysis of reduced *γ*-tocopherol content in Ethiopian mustard.

## 2. Material and Methods

### 2.1. Plant Materials

Ethiopian mustard line BCT-6 is characterized by a reduced accumulation of *γ*-tocopherol, which results in an increased relative concentration of *α*-tocopherol and a reduced total tocopherol content [11]. The authors reported a tocopherol content of 127 mg kg^−1^ and *α*-/*γ*-tocopherol ratio of 3.07 in BCT-6 compared to 185 mg kg^−1^ and *α*-/*γ*-tocopherol ratio of 1.17 in the control line C-101.

### 2.2. Genetic Study

Plants of C-101 used as female were crossed with plants of BCT-6 in 2009. F_1_ plants were grown in pots under open-air conditions together with plants of both parents in 2010. F_1_ plants were bagged for self-fertilization and also backcrossed to both parents, which were used as female plants in the crosses. In November 2012, forty-eight seeds of the parents and F_1_, 96 BC_1_F_1_ seeds to both parents, and 240 F_2_ seeds were placed in moistened filter papers in Petri dishes for germination and sown in small pots 7 × 7 × 7 cm filled with a commercial substrate. The plants were maintained in a growth chamber for three weeks at 25°C day and 20°C night with 16 h photoperiod and then transplanted into the field following a randomized block design with three replications, each replication containing one-third of the plants. Plants were arranged in rows 5 m long with a separation of 1 m between rows and 15 cm between plants in the row. Before the first flowers opened, 2-3 central branches were bagged with microperforated plastic bags made of SM570Y film (Cryovac, Sealed Air Corporation, Elmwood Park, NJ, USA) to produce seeds from self-fertilization. Bags from individual plants were harvested in July 2012. Tocopherols were analyzed quantitatively in bulked seeds of each plant shortly after harvest.

### 2.3. Analysis of Tocopherols

Around 100 mg of bulked seeds from single plants was used for tocopherol analyses. After weighing, the seeds were crushed as fine as possible with a stainless steel rod and tocopherols were extracted with 4 mL isooctane. The tubes were vortexed and maintained in the dark overnight, after which they were vortexed again and centrifuged. After filtering, tocopherols were analyzed by high-performance liquid chromatography (HPLC) as described previously [22], using isooctane/tert-butylmethylether (94 : 6) at a flow rate of 0.8 mL/min as eluent and fluorescence detection (Waters 474) at 295 nm excitation and 330 nm emission. The column was a Lichrospher diol, 250 mm × 2 mm internal diameter (Merck Millipore, Darmstadt, Germany), connected to a silica guard column Lichrospher Si 60, 4 mm × 4 mm internal diameter (Merck Millipore). External calibration curves were developed for each of the tocopherol homologues *α*-, *γ*-, and *δ*-tocopherol using a tocopherol standard set (Calbiochem, Merck Millipore). Tocopherols were expressed as mg kg^−1^ air-dried seeds.

### 2.4. Statistical Analyses

Generation means analysis was conducted on *γ*-tocopherol content and total tocopherol content to test whether their genetic control can be explained by additive-dominance models or, alternatively, whether epistatic or nonallelic interactions may be influencing the traits. Individual scaling tests *A*, *B*, and *C* were first conducted to identify presence or absence of epistatic interactions. They were defined as [23]

(1) A=2B−c1.1−P−1−F−1,B=2B−c1.2−P−2−F−1,C=4F−2−2F−1−P−1−P−2,

where ${\overset{-}{P}}_{1},\;\;{\overset{-}{P}}_{2},\;\;{\overset{-}{F}}_{1},\;\;{\overset{-}{F}}_{2},\;\;{\overset{-}{B}c}_{1.1},\;\;and\;\;{\overset{-}{B}c}_{1.2}$ are the observed mean values of the two parents lines BCT-6 and C-101 and the F_1_, F_2_, and BC_1_ to both parents, respectively.

When no epistatic interactions were identified, a three-parameter joint scaling test was conducted to confirm the model and to test its goodness of fit. If epistatic interactions were detected, Cavalli's six-parameter joint scaling test was conducted to characterize additive × additive, additive × dominant, and dominant × dominant interactions [21]. The minimum number of genes (*k*) controlling total tocopherol content was estimated using the Castle-Wright equation [24]:

(2) $$\begin{array}{c} k=\frac{{\left(P_{1}-P_{2}\right)}^{2}}{8\left[{\sigma_{}^{2}}_{F2}-\left({\sigma_{}^{2}}_{P1}+{\sigma_{}^{2}}_{P2}+{\sigma_{}^{2}}_{F1}\right)/3\right]}, \end{array}$$

where P_1_ and P_2_ are the mean values of the two parents and *σ* ^2^_P1_, *σ* ^2^_P2_, *σ* ^2^_F1_, and *σ* ^2^_F2_ are the variances of the parents and F_1_ and F_2_ generations. Correlation coefficients, matrix inversions, and any other auxiliary computation were conducted using IBM SPSS Statistics v. 23.

## 3. Results

Table 1 shows the *α*-, *γ*-, and total tocopherol contents of the six generations evaluated in the study. Differences between both parents were of small magnitude for *α*-tocopherol content (86.57 mg kg^−1^ in C-101 and 70.04 mg kg^−1^ in BCT-6), whereas larger differences were observed for *γ*-tocopherol content (75.52 mg kg^−1^ in C-101 and 23.01 mg kg^−1^ in BCT-6) and total tocopherol content (167.17 mg kg^−1^ in C-101 and 94.49 mg kg^−1^ in BCT-6). Because of the small differences between the parents for *α*-tocopherol content, the genetic study focused only on *γ*-tocopherol and total tocopherol contents. For both traits, the average values of F_1_ (56.82 mg kg^−1^ for *γ*-tocopherol, 134.58 mg kg^−1^ for total tocopherol content) were slightly higher than the mid parent value (49.27 mg kg^−1^ for *γ*-tocopherol, 130.83 mg kg^−1^ for total tocopherol content), suggesting incomplete dominance of alleles from C-101.

**Figure 1:**
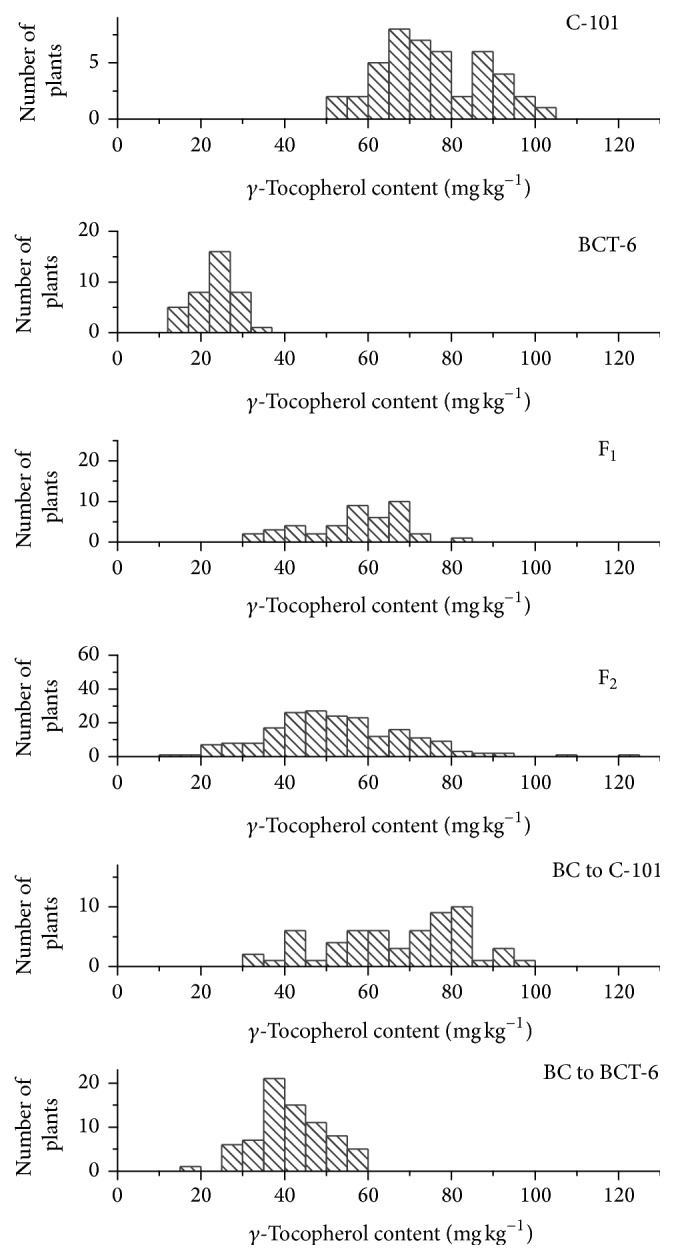
Histograms of *γ*-tocopherol content (mg kg^−1^ seed) in the Ethiopian mustard lines C-101 and BCT-6 and the F_1_, F_2_, and BC generations derived from their cross.

**Figure 2:**
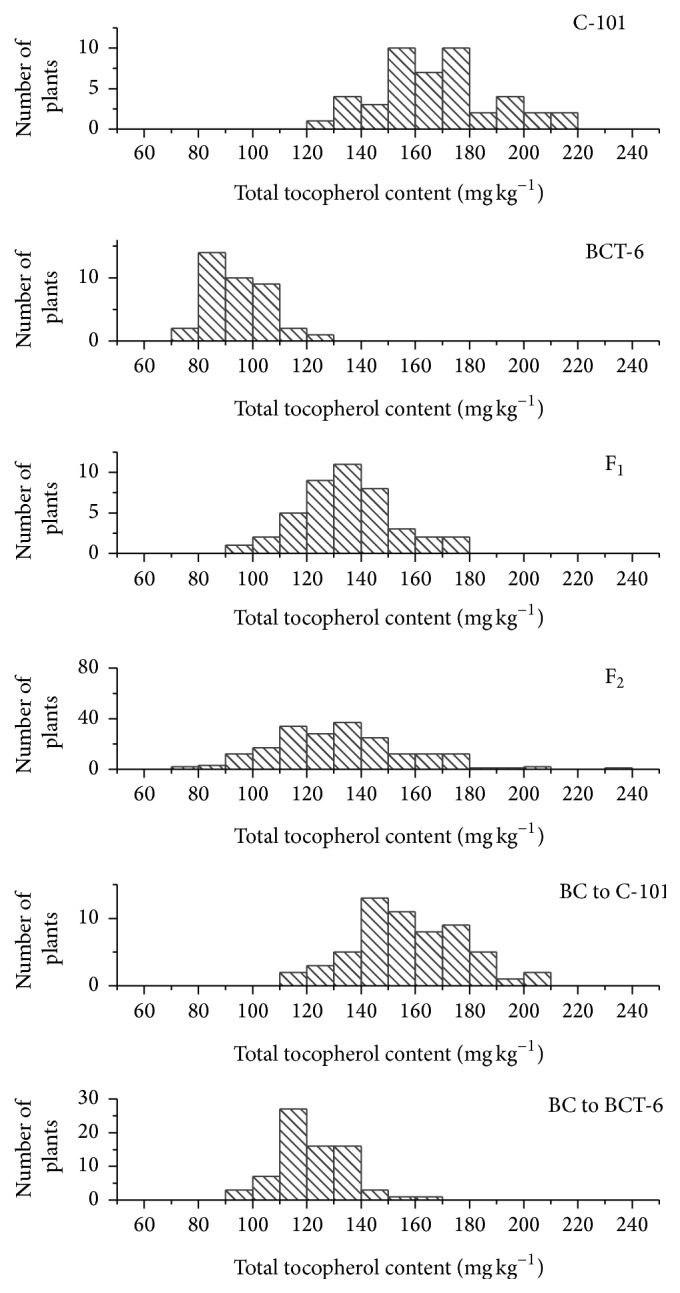
Histograms of total tocopherol content (mg kg^−1^ seed) in the Ethiopian mustard lines C-101 and BCT-6 and the F_1_, F_2_, and BC generations derived from their cross.

Histograms for *γ*-tocopherol content in the six generations are shown in Figure 1. The ranges of variation of both parents did not overlap, ranging from 13.1 to 36.1 mg kg^−1^ in BCT-6 and from 50.7 to 104.8 mg kg^−1^ in C-101. In the case of total tocopherol content, there was only a small overlapping area in the ranges of variation of both parents (Figure 2).

Individual scaling tests *A*, *B*, and *C* were not significant for *γ*-tocopherol content (Table 2), indicating absence of epistasis and accordingly genetic control by additive and dominant components. Conversely, both *A* and *B* tests were significant for total tocopherol content (Table 2), suggesting the presence in this case of epistatic interactions.

A three-parameter joint scaling test was then computed for *γ*-tocopherol content to confirm the model and to test its statistical significance, whereas a six-parameter model, including interactions, was computed for total tocopherol content. The estimates of the mean [m], additive [a], and dominant [d] components and digenic interactions additive × additive [aa], additive × dominant [ad], and dominant × dominant [dd] are shown in Table 3. In the case of *γ*-tocopherol content, both the additive and dominant components were significant, with predominance of the additive component. For total tocopherol content, there was also predominance of the additive component but in this case all digenic interactions were significant at *P* < 0.05, although additive × additive and dominant × dominant interactions were not significant at *P* < 0.01 (Table 3). The fact that dominant and dominant × dominant components had opposite sign may indicate the presence of duplicate interactions [21].

For total tocopherol content, the joint scaling test comprised six genetic parameters and six generations, which let no degrees of freedom for testing the goodness of fit of the model. However, the existence of three degrees of freedom allowed the goodness of fit of the model to be tested for *γ*-tocopherol content. Observed and expected values for each generation, computed using the estimates of [m], [a], and [d], are presented in Table 4. Chi-square test confirmed the adequacy of the additive-dominance model for the genetic control of reduced *γ*-tocopherol content in BCT-6.

The *k* estimate for minimum number of genes resulted in values of 1.94 for *γ*-tocopherol content and 1.96 for total tocopherol content, suggesting that the reduced *γ*-tocopherol and total tocopherol contents in BCT-6 might be oligogenic rather than polygenic traits.

**Figure 3:**
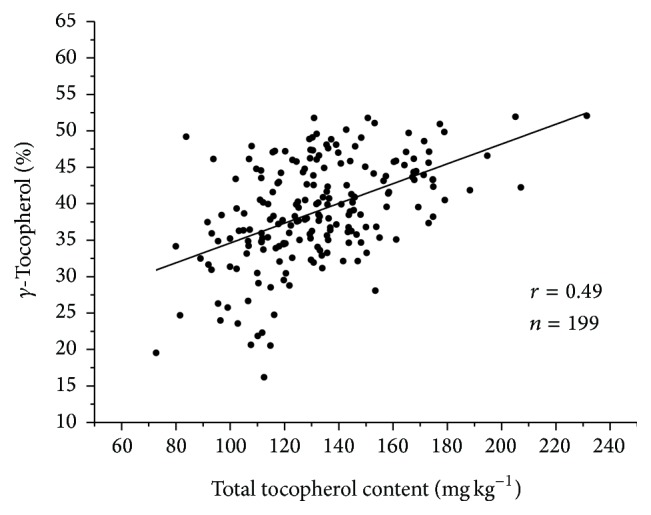
Scatter plot of total seed tocopherol content (mg kg^−1^ seed) and concentration of *γ*-tocopherol (% total tocopherols) in a population of 199 F_2_ plants from the cross between the Ethiopian mustard lines BCT-6, with reduced *γ*-tocopherol content, and C-101, with conventional levels of *γ*-tocopherol.

A positive correlation between *α*-tocopherol content and *γ*-tocopherol content was observed in the F_2_ generation (Table 5). Both traits were in turn positively correlated with total tocopherol content. When considering additionally the relative concentration of each tocopherol form, expressed as percentage of total tocopherols, it was observed that the percentage of *γ*-tocopherol showed a highly positive correlation with *γ*-tocopherol content (*r* = 0.83, *P* < 0.01), while the correlation between the relative concentration of *α*-tocopherol and *α*-tocopherol content was positive but of small magnitude (*r* = 0.17, *P* < 0.05). Total tocopherol content was negatively correlated with the relative concentration of *α*-tocopherol (*r* = −0.49) and positively correlated with the relative concentration of *γ*-tocopherol (*r* = 0.49). The relationship between total tocopherol content and concentration of *γ*-tocopherol of the F_2_ plant generation is shown in Figure 3.

## 4. Discussion

Reduced accumulation of *γ*-tocopherol in the seeds of Ethiopian mustard line BCT-6 is a unique trait that has not been reported in other nontransgenic germplasm of species of the genus* Brassica*. In* B. juncea*, reduced *γ*-tocopherol proportion with no significant changes in total tocopherol content has been obtained by overexpression of a *γ*-TMT, which converts *γ*-tocopherol into *α*-tocopherol [13]. The results of the present research suggested that the trait is under the control of a low number of genes that follow a dominance-additive model with no significant epistatic interactions, with predominance of additive effects. Previous studies on the genetic control of tocopherol content in rapeseed concluded also predominance of additive effects [14–16]. The absence of nonallelic or epistatic interactions has important implications for the management of this trait in breeding programs, since such interactions can hinder the identification of the target genotypes and also can be an obstacle in genetic mapping [25]. Contrasting results in the estimates of the heritability of individual and total tocopherol content have been reported in rapeseed, probably because of the use of different genetic material [17–20]. In the present research, heritability was not estimated because the line with reduced *γ*-tocopherol content had been selected for this trait during several generations. It was selected from individual plants that exhibited the mutant trait within a germplasm accession with an average tocopherol profile close to the wild type [11]. The estimation of the minimum number of genes suggested that the trait is likely to be controlled by only two genes which, together with the absence of epistatic effects, may anticipate good response to selection.

BCT-6 was developed through pedigree selection in a program in which germplasm with opposite tocopherol profile, that is, with very high relative concentration of *γ*-tocopherol, was also selected [11]. The latter trait was found to be controlled by a single gene, allegedly a *γ*-TMT that converts *γ*-tocopherol into *α*-tocopherol. Loss of function of *γ*-TMT has resulted in nearly complete replacement of *α*-tocopherol by *γ*-tocopherol in sunflower [26] and safflower [27]. The opposite situation, that is, increased *α*-tocopherol and reduced *γ*-tocopherol relative concentrations, has been reported to occur due to higher activity of the *γ*-TMT promoter in soybean [28], to the presence of additional active copies of the *γ*-TMT gene in sunflower [29], and also to induction by overexpression of the *γ*-TMT gene in several species such as* Arabidopsis*, soybean, and* Brassica juncea* [13, 30–32]. The Ethiopian mustard line BCT-6 showed a reduction of total *γ*-tocopherol content in addition to a change in the relative proportion of *α*- and *γ*-tocopherol, which has not been reported in any of the abovementioned studies. The genetic basis of the BCT-6 phenotype is unknown and might be more complex than a shifted ratio from *γ*- to *α*-tocopherol due to a *γ*-TMT alteration. Other mechanisms of reduction of *γ*-tocopherol content in seeds have been proposed. In* Arabidopsis*, overexpression of tocopherol biosynthesis genes such as *γ*-TMT [30], tocopherol cyclase, and homogentisate phytyl transferase [33] from rapeseed, a species closely related to Ethiopian mustard, resulted also in reduced accumulation of *γ*-tocopherol in the seeds.

Reduction of *γ*-tocopherol content in BCT-6 resulted in lower levels of total tocopherol content as shown by a highly significant positive correlation (*r* = 0.89) between both traits. Although the traits were highly correlated, differences in their genetic control, such as the existence of epistatic effects influencing total tocopherol content, were found. Previous studies [16, 20] found similar high correlation coefficients (0.92 and 0.91, resp.) between both traits in segregating populations of F_1_-derived doubled haploid rapeseed lines. Those studies reported the existence of common QTL regions controlling *γ*- and total tocopherol content, but also other independent QTL with main additive effects only affecting *γ*-tocopherol content. Additionally, a positive correlation between *α*- and *γ*-tocopherol contents has been found in this study in the F_2_ generation. In general, previous studies on rapeseed have detected no significant correlations between accumulations of both tocopherol forms [15, 17, 19, 34, 35], although other studies [18, 20] also found a significant positive correlation (0.51 and 0.29, resp.) between both traits in segregating doubled haploid populations. We also observed a positive correlation between total tocopherol content and the relative concentration of *γ*-tocopherol, which has been reported in some previous studies on Ethiopian mustard [11] and rapeseed [15, 17, 20], but other studies reported no significant correlation between both traits [19, 33]. It will be therefore important to study such correlations in different environments and genetic backgrounds to evaluate the feasibility of developing Ethiopian mustard germplasm with high total tocopherol content coupled with a tocopherol profile dominated by *α*-tocopherol.

Amongst the four tocopherol forms, *α*-tocopherol has the maximum* in vivo* vitamin E activity. It has been estimated that *γ*-tocopherol possesses just around 10% of the vitamin E activity of *α*-tocopherol [4]. Vitamin E is the major lipophilic antioxidant in the human body, playing a major role in reducing oxidative damage and subsequently mitigating aging effects and chronic diseases associated with oxidative stress [36]. Two major vegetable oils, sunflower and olive, contain nearly all the tocopherols (>90%) in the *α*-tocopherol form. Conversely, rapeseed/canola oil contains on average around 40% of the tocopherols in the *α*-tocopherol form [37]. The development of commercial Ethiopian mustard oil with the high relative concentration of *α*-tocopherol found in line BCT-6, around 70%, may have interest for uses such as salad oils or margarines, particularly in areas where the intake of vitamin E is below the recommended dietary allowances [38]. This will require characterizing previously the genetic alteration in BCT-6 that results in high relative concentration of *α*-tocopherol and determining whether the concomitant reduction in total tocopherol content observed in BCT-6 seeds can be overcome in selected progeny from this line.

## 5. Conclusions

The present research concluded that reduced accumulation of *γ*-tocopherol and subsequent increased proportion of *α*-tocopherol in the seeds of Ethiopian mustard line BCT-6 follow a dominant-additive genetic model with predominance of additive effects. The minimum number of genes controlling the trait was estimated in two. This anticipates good response to selection in breeding programs. Reduced concentration of *γ*-tocopherol was associated with reduced total tocopherol content. The feasibility of overcoming such a correlation should be investigated in order to develop germplasm containing high levels of tocopherols in the seeds with a profile dominated by *α*- instead of *γ*-tocopherol.

## Figures and Tables

**Table 1:** *α*-Tocopherol, *γ*-tocopherol, and total tocopherol contents (mean ± variance, in mg kg^−1^ air-dried seed) in the Ethiopian mustard lines C-101 and BCT-6 and the F_1_, F_2_, and BC generations derived from their cross.

Generation	*n*	*α*-Tocopherol	*γ*-Tocopherol	Total tocopherol
		mg kg^−1^		
C-101	45	86.57 ± 140.84	75.52 ± 169.74	167.17 ± 505.26
BCT-6	38	70.04 ± 78.94	23.01 ± 24.81	94.49 ± 134.94
F_1_	43	74.39 ± 128.30	56.82 ± 129.86	134.58 ± 284.67
F_2_	199	76.37 ± 173.35	52.69 ± 291.11	132.25 ± 638.53
BC to C-101	59	86.87 ± 106.59	67.12 ± 251.86	158.06 ± 428.83
BC to BCT-6	74	78.03 ± 40.74	41.62 ± 67.34	122.07 ± 158.46

**Table 2:** Individual scaling tests *A*, *B*, and *C* for *γ*-tocopherol content and total tocopherol content in the cross between the Ethiopian mustard lines BCT-6, with reduced *γ*-tocopherol content, and C-101, with conventional levels of *γ*-tocopherol.

Test	*γ*-Tocopherol content	Total tocopherol content
*A*	1.90 ± 4.89^ns^	14.37 ± 6.85^*∗*^
*B*	3.41 ± 2.70^ns^	15.07 ± 4.33^*∗∗*^
*C*	−1.41 ± 6.31^ns^	−1.82 ± 9.61^ns^

*∗* and *∗∗*: significant at *P* < 0.05 and *P* < 0.01 probability levels, respectively.

ns = not significant.

**Table 3:** Estimates of additive, dominance, and digenic interaction parameters (± standard error) for *γ*-tocopherol content and total tocopherol content through a joint scaling test in the cross between the Ethiopian mustard lines BCT-6, with reduced *γ*-tocopherol content, and C-101, with conventional levels of *γ*-tocopherol. *t*-values for significance tests are given in parentheses.

Test	*γ*-Tocopherol content	Total tocopherol content
m	49.26 ± 0.95^*∗∗*^ (*t* = 51.85)	110.09 ± 9.84^*∗∗*^ (*t* = 11.19)
[a]	25.97 ± 0.93^*∗∗*^ (*t* = 27.92)	39.00 ± 1.92^*∗∗*^ (*t* = 20.31)
[d]	8.58 ± 1.75^*∗∗*^ (*t* = 4.90)	64.94 ± 24.71^*∗∗*^ (*t* = 2.63)
[aa]		22.22 ± 9.63^*∗*^ (*t* = 2.31)
[ad]		−19.78 ± 7.27^*∗∗*^ (*t* = −2.72)
[dd]		−40.35 ± 15.91^*∗*^ (*t* = −2.54)

*∗* and *∗∗*: significant at *P* < 0.05 and *P* < 0.01 probability levels, respectively.

ns = not significant.

**Table 4:** Goodness of fit of the additive-dominance model for *γ*-tocopherol content in the cross between the Ethiopian mustard lines BCT-6, with reduced *γ*-tocopherol content, and C-101, with conventional levels of *γ*-tocopherol.

Generation	Observed (*O*)	Expected (*E*)	Weight (*W*)^a^	(*O* − *E*)^2^ × *W*
C-101	75.52	75.23	0.2653	0,0223
BCT-6	23.01	23.30	1.5385	0,1294
F_1_	56.82	57.85	0.3311	0,3513
F_2_	52.69	53.56	0.6849	0,5184
BC to C-101	67.12	66.54	0.2342	0,0788
BC to BCT-6	41.62	40.52	1.0989	1,3297

*χ* ^2^ (*P*)				2.43 (0.18)

^a^Weights are obtained in the joint scaling test (Hill et al., 1998 [21]) shown in Table 3.

**Table 5:** Correlation coefficients between *α*-tocopherol content (*α*-T), *γ*-tocopherol content (*γ*-T), and total tocopherol content (total-T), expressed as mg kg^−1^ seed, and relative concentrations of *α*-tocopherol ([*α*-T]) and *γ*-tocopherol ([*γ*-T]), expressed as percentage of the total tocopherols.

	*γ*-T	Total-T	[*α*-T]	[*γ*-T]
*α*-T	0.38^*∗∗*^	0.77^*∗∗*^	0.17^*∗*^	−0.17^*∗*^
*γ*-T		0.89^*∗∗*^	−0.82^*∗∗*^	0.83^*∗∗*^
Total-T			−0.49^*∗∗*^	0.49^*∗∗*^
[*α*-T]				−0.99^*∗∗*^

*∗* and *∗∗*: significant at *P* < 0.05 and *P* < 0.01 probability levels, respectively.

## Abbreviation

*γ*-TMT: *γ*-Tocopherol methyltransferase.
